# Growth performance, carcass yield and characteristics, meat quality, serum biochemistry, jejunal histomorphometry, oxidative stability of liver and breast muscle, and immune response of broiler chickens fed natural antioxidant alone or in combination with *Bacillus licheniformis*

**DOI:** 10.5194/aab-65-183-2022

**Published:** 2022-05-09

**Authors:** Umair Ahsan, Shahram Golzar Adabi, Özge Sayın Özdemir, Ömer Sevim, Onur Tatlı, Eren Kuter, Özcan Cengiz

**Affiliations:** 1 Department of Plant and Animal Production, Burdur Vocational School of Food, Agriculture and Livestock, Burdur Mehmet Akif Ersoy University, İstiklal Campus, Burdur 15030, Turkey; 2 Centre for Agriculture, Livestock and Food Research, Burdur Mehmet Akif Ersoy University, İstiklal Campus, Burdur 15030, Turkey; 3 Huvepharma, Istanbul 6144, Turkey; 4 Department of Animal Nutrition and Nutritional Diseases, Faculty of Veterinary Medicine, Aydın Adnan Menderes University, Işıklı, Aydın 09016, Turkey; 5 Department of Animal Nutrition and Nutritional Diseases, Faculty of Veterinary Medicine, Burdur Mehmet Akif Ersoy University, İstiklal Campus, Burdur 15030, Turkey

## Abstract

In this study, oxidative stability of liver and breast meat, and immune response were evaluated in broiler chickens fed supplemental phytogenic feed additive (PFA) alone or in combination with *Bacillus licheniformis*. Three experimental groups – control, PFA (60 mg kg ${}^{-1}$ ), and PFA (60 mg kg ${}^{-1}$ ) + 0.5 mg kg ${}^{-1}$ *B. licheniformis* (1.6 × 10 ${}^{12}$ cfu g ${}^{-1}$ ), each consisting of 5 replicates – were established with 20 one-day-old chickens per replicate (300 birds in total). Growth performance, carcass yield and characteristics, and meat quality remained unaffected. However, supplemental PFA and PFA + *B. licheniformis* improved the serum biochemistry and jejunal histomorphometry of broiler chickens ( P<0.05 ). PFA and PFA + *B. licheniformis* groups had lower thiobarbituric acid reacting substances (TBARS) in liver, and freeze–thaw breast meat after 30, 60, and 90 d of storage ( P<0.05 ). PFA and PFA + *B. licheniformis* supplementation lowered the carbonyl group in fresh and stored breast meat ( P<0.05 ). Antibody titer against infectious bursal disease virus was higher in the PFA + *B. licheniformis* group than the control group ( P<0.05 ). It can be concluded that PFA or PFA + *B. licheniformis* in broiler diets improves the health, oxidative stability of liver and breast meat, and immune response of broiler chickens.

## 1 Introduction

Several alternatives have been under investigation to promote the antibiotic-free poultry production following the ban on subtherapeutic use of antibiotics in animal diets due to declining antibiotic efficacy in human medicine attributed to cross-resistance against antibiotics in microbes. In practice, the alternative growth-promoting agents should play the same role as subtherapeutic doses of antibiotics in animal diets. Probiotics (direct-fed microbials), prebiotics (oligosaccharides), nutrients (fatty acids, amino acids, vitamins, and minerals), and whole, parts, or derived bioactive molecules of plants, herbs, and spices (phytogenic feed additives; PFAs) are among these alternatives (Das et al., 2012). These substances have shown growth-promoting properties in response to dietary supplementation to replace the use of antibiotics (Jamroz et al., 2005; Das et al., 2012; Ahsan et al., 2016; Chacher et al., 2017). PFA, derived from botanical sources, may consist of whole or parts of plants, herbs, or spices, extracts (aqueous, or alcoholic), and/or the essential oils or oleoresins comprising of bioactive molecules of the botanical source (Yitbarek, 2015). Apart from growth-promoting effects, PFA have been shown to improve the nutrient digestibility (Malayoğlu Basmacioğlu et al., 2010; Paraskeuas et al., 2017), intestinal morphometry and microbiota (Wlodarska et al., 2015; Wati et al., 2015; Ahsan et al., 2018), immunity (Kim et al., 2013; Lu et al., 2014), and antioxidant activity (Ciftci et al., 2010). *Bacillus licheniformis*, a direct-fed microbial or probiotic, has lately gained attention in poultry nutrition. Previous studies have reported that dietary supplementation of *B. licheniformis* improves the growth performance (Zhou et al., 2016), gut microbiota (Xu et al., 2018), intestinal morphology, and cecal volatile fatty acid production (Musa et al., 2019).

Due to the enrichment of poultry diets with polyunsaturated fatty acids (PUFAs) to provide essential fatty acids through chicken meat and eggs (indispensable for animal health as for public health), synthetic antioxidants have been a compulsory component of animal diets. Antioxidants prevent the rancidity attributable to lipid peroxidation (especially PUFAs) and to improve the oxidative stability of complete feeds during processing, storage, and feed out stages in addition to that of meat (Salami et al., 2015). Nonetheless, synthetic antioxidants have been characterized as carcinogenic, thus attracting the use of safer antioxidants. Consequently, natural antioxidants are preferred over their synthetic counterparts. Natural antioxidants are usually labeled “generally regarded as safe” (GRAS). PFAs are known to contain bioactive compounds that exhibit antioxidant properties through various pathways (Salami et al., 2016). However, PFA is a broader term that encompasses all the additives from botanical sources. Therefore, a wide range of PFAs are available comprising of single or multiple botanical sources that differ in their composition and bioactive components. Accordingly, inconsistent results have been reported regarding the effectiveness of PFA, thus requiring optimization in terms of their selection and usage regimens. Mountzouris et al. (2015) suggested the use of probiotic along with PFA to overcome this issue. Hence, we hypothesized that dietary PFA in combination with *B. licheniformis* may prevent the lipid and protein oxidation of fresh and freeze–thaw breast meat that might improve the growth performance, carcass yield and characteristics, serum biochemistry, meat quality, and jejunal histomorphometry of broiler chickens. In this study, the group involving the single use of dietary *B. licheniformis* was not employed since previous ones have reported the use of *B. licheniformis* alone in diets of broiler chickens (Knap et al., 2010; Zhou et al., 2016; Xu et al., 2018; Musa et al., 2019; Zhao et al., 2020). The PFA used in our study is characterized by capsaicin, glucosinolate, saponins, terpenes, and curcumin. To the best of our knowledge, no previous study has reported the use of such PFA alone or in combination with *B. licheniformis* in broiler diets. Therefore, we assessed the growth performance, carcass yield and characteristics, serum biochemistry and lipid peroxidation, jejunal histomorphometry, meat quality, and lipid and protein oxidation of fresh and freeze–thaw breast meat of broiler chickens fed PFA alone or in combination with *B. licheniformis*.

## 2 Materials and methods

The experiment was steered at the poultry research facility of Aydın Adnan Menderes University, Turkey. All the procedures were consistent with the guidelines of a local ethical committee for the use of animals in experimental studies (approval no. 64583101/2020/091).

### 2.1 Study design and experimental groups

The study design was completely randomized in which 300 one-day-old Ross 308 male broiler chickens (Egetav Tavukçuluk San. ve Tic. A.Ş., İzmir, Turkey) were randomly distributed to 3 experimental groups each having 5 floor pens as replicates with 20 birds in each pen. All the groups received basal diets for starter, grower, and finisher phases (Table 1) based on recommended nutrient specifications by Aviagen (2019). The control group remained untreated throughout the experiment receiving diets without supplementation. Other groups received dietary supplementation of 60 mg kg ${}^{-1}$ PFA characterized by 573 µ g g ${}^{-1}$ capsaicin (15 % *Capsicum annuum* L. var. *minimum* (Miller) Heiser/hot pepper extract), 26.9 g kg ${}^{-1}$ glucosinolates (15 % *Sinapis alba L.*/white mustard extract), 29.3 g kg ${}^{-1}$ saponins (25 % *Saponaria officinalis L.*/soapwort extract), 65.2 g kg ${}^{-1}$ terpenes (25 % *Acorus calamus* L./sweet flag extract), and 11.4 g kg ${}^{-1}$ curcumin (15 % *Curcuma longa L.*/turmeric extract) alone or in combination with 0.5 g kg ${}^{-1}$ of *B. licheniformis* DSM 28710 (3.2 × 10 ${}^{9}$ cfu g ${}^{-1}$ ) as a single-strain probiotic.

**Table 1:** Composition of basal diets for different growth phases (g kg ${}^{-1}$ , as feed basis).

Ingredients	Starter (day 0–10)	Grower (day 11–24)	Finisher (day 25–42)
Corn	516.42	443.74	407.22
Soybean meal	289.21	187.40	76.54
Full-fat soybean	123.76	160	260
Barley	–	100	150
Wheat bran	40	40	40
Meat and bone meal	–	26.62	26.81
Soy oil	–	21.62	19.99
Limestone	10.9	7.5	7.14
Dicalcium phosphate	4.63	–	–
Salt	1.01	0.650	0.55
Sodium bicarbonate (NaHCO ${}_{3}$ )	2.53	1.5	1.6
Methionine hydroxy analogue	3.44	3.32	2.99
L-Lysine sulfate	3.26	2.93	2.59
L-Threonine	1	0.74	0.52
Vitamin and mineral premix ${}^{1}$	2	2	2
Enzyme premix (phytase + NSPase) ${}^{2}$	1	1	1
Anticoccidial ${}^{3}$	0.5	0.5	0.5
Choline chloride	0.34	0.48	0.55
Nutrient content ${}^{4}$			
ME, MJ/kg	12.34	12.97	13.26
Crude protein, %	23 (22.88)	21 (21.14)	19.5 (19.63)
Crude fiber, %	3.92 (3.62)	4.20 (3.98)	4.02 (3.91)
Crude ash, %	4.85 (5.11)	4.88 (5.04)	4.85 (4.96)
Crude fat, %	4.84 (4.78)	7.91 (6.46)	9.55 (8.92)
Ca, %	0.96	0.87	0.78
avP, %	0.48	0.435	0.39
Digestible methionine, %	0.59	0.54	0.51
Digestible methionine + cysteine, %	0.93	0.85	0.78
Digestible lysine, %	1.28	1.12	1.02

${}^{1}$ Supplied the following per kilogram of complete feed: 4.13 mg retinyl acetate; 125 µ g cholecalciferol; 100 mg DL- α -tocopheryl acetate; 3.5 mg menadione; 3.5 mg thiamin; 9 mg riboflavin; 20 mg calcium D-pantothenate; 65 mg niacin; 0.02 mg vitamin B12; 2.2 mg folic acid; 4.5 mg pyridoxine; 0.22 mg biotin; 120 mg manganese (Mn oxide); 1.5 mg iodine (Ca iodate); 25 mg iron (Fe sulfate); 16 mg copper (Cu sulfate); 110 mg zinc (Zn oxide); 0.3 mg selenium (Na selenite). ${}^{2}$ OptiPhos^®^ + Hostazym^®^ X (Huvepharma^®^ EOOD, Sofia, Bulgaria). ${}^{3}$ Sacox^®^ (Huvepharma^®^ EOOD, Sofia, Bulgaria). ${}^{4}$ Values given inside parentheses indicate analyzed results, whereas values outside the parentheses refer to calculated.

### 2.2 Rearing management

Floor pens were installed in the experimental room measuring 1 m × 1 m floor space available for the chickens which excluded the space occupied by drinkers and feeders. The birds were reared in a deep litter system, and an 8 cm deep layer of wood shavings was spread in each floor pen. Automatic heaters and fans were used to maintain the temperature and relative humidity. Experimental room was pre-heated to 32 ${}^{\circ }$ C that was maintained in the first days of the experiment later reduced by 3.5 ${}^{\circ }$ C per week until day 21 of the experiment. Subsequently, the temperature was maintained between 24 and 26 ${}^{\circ }$ C until the end of experiment. Ad libitum provision of water and feed was warranted using three nipple drinkers and one bell feeder in each pen. Lighting program was implemented according to the recommendations of management manual for Ross 308 broiler chickens. Diets were switched to starter, grower, and finisher phases during days 0–10, 11–24, and 25–42, respectively. After the slaughtering procedure at day 24, all the remaining birds were vaccinated against infectious bursal disease (IBD) by Nobilis Gumboro 228E (MSD Animal Health) via drinking water.

### 2.3 Growth performance

Chickens were individually weighed at the time of arrival, 10, 24, and 42 d of experiment. The quantities of feeds were distributed, and leftovers were weighed in each phase. Body weight (BW) gain and feed intake (FI) were calculated through difference method. Mortalities were recorded during the experiment to adjust the feed conversion ratio (FCR), which was calculated using a standard formula of ratio between feed intake and weight gain.

### 2.4 Slaughtering procedure

The birds were subjected to overnight fasting ( ∼ 6 h) for slaughtering at day 42. At the end of grower (day 24) and finisher phases (day 42), four birds close to average body weight of pen were randomly selected, slaughtered by decapitation, and the decapitated birds were left for complete exsanguination. Subsequently, softening of feathers was accomplished at 57 ${}^{\circ }$ C for 2 min using an electric scalding device (Cimuka Kuluçka Makinaları, Ankara, Turkey) followed by removal of feathers in feather removing equipment (Cimuka Kuluçka Makinaları, Ankara, Turkey). Finally, the carcasses were carefully dissected for further sampling and analyses.

### 2.5 Carcass yield and characteristics

Measurement of carcass yield and characteristics were carried out at day 42 only. Hot carcasses, liver, breast, and thigh were weighed. Carcass and liver yields were calculated relative to the slaughter weight. Breast and thigh yields were calculated relative to the hot carcass weights.

### 2.6 Jejunal histomorphometry

Histomorphometry of jejunum was carried out at day 24. For this purpose, the abdomens were opened, intestines were removed, and 3 cm of jejunal samples was dissected from the midpoint of jejunum towards proximal direction (towards duodenum) after the identification of Meckel's diverticulum as a reference point. The jejunal tissues were immediately washed, immersed in 10 % neutral buffered formalin for fixation, soaked in ascending alcohol concentrations (70 %, 80 %, 96 %, and 100 %) for dehydration, cleared in xylene, and finally embedded in paraffin blocks. Sections were obtained, stained with hematoxylin and eosin and periodic acid–Schiff, and examined under light microscope (BX51, Olympus, Japan), and images were produced using a digital camera (SC180, Olympus, Japan). Villus height, villus diameter, and villus width were measured using a computer-assisted image analysis program (Leica QWin Standard, Version 2.8, Germany) following the procedures previously described by Ahsan et al. (2018). Villus height to crypt depth ratio was measured by division method, whereas villus surface area was computed according to de los Santos et al. (2005) using following Eq. (1):

1 $$\begin{array}{rl} \text{Villus surface area} & =2\pi \;\times \;\left(\text{villus width}/2\right)\\ & \times \;\left(\text{villus height}/10^{6}\right). \end{array}$$

### 2.7 Serum biochemistry

Prior to the slaughtering at day 24 and day 42 of the experiment, blood samples were collected from the tibial veins of birds (four birds per replicate) by venepuncture in serum vacutainers with gel as clotting activator. After clotting, sera were separated by centrifugation at 4500 rpm for 15 min. Sera were separated into Eppendorf tubes in duplicates for biochemical and serological analyses.

Serum total protein (TP), albumin, aspartate transaminase (AST), alanine transaminase (ALT), and gamma-glutamyltransferase (GGT) levels were measured following colorimetric and kinetic spectrophotometric method using commercial kits (Randox RX series, Randox Laboratories Ltd., Crumlin, United Kingdom). The samples were prepared according to the procedure outlined in the manufacturer's manual using reagents in the commercial kits followed by reading of values in an automatic clinical chemistry analyzer (Randox RX Monaco, Randox Laboratories Ltd., Crumlin, United Kingdom).

### 2.8 Meat quality attributes

Attributes of breast meat quality were measured at the time of slaughter and 24 h postmortem at day 42 of experiment. Breast fillets were collected and cooled at 4 ${}^{\circ }$ C immediately after dissection and weighing of breast fillets. Following the measurement of pH and color at slaughter, breast fillets were stored at 4 ${}^{\circ }$ C for 24 h post-slaughter measurements. pH of breast fillets was measured after cooling (at slaughter and 24 h post-slaughter) by inserting the probe of a waterproof portable pH meter (Testo 205, Testo Inc., Lenzkirch, Germany) into the cranial, middle, and caudle 1/3 portions of the pectoralis major muscle, and the mean was taken as the final measurement. Measurement of meat color was accomplished at slaughter and 24 h postmortem in terms of lightness (L ${}^{*}$ ), redness (a ${}^{*}$ ), and yellowness (b ${}^{*}$ ) according to the recommendations of Commission Internationale de l'Eclairage (CIE; International Commission on Illumination) with the help of a chromameter (Minolta CR400; Konica Minolta Sensing Inc., Osaka, Japan). Compression method (Barton-Gade et al., 1993) was employed to measure the drip loss of breast meat (24 h postmortem). Briefly, 5 g (approximately) breast meat was finely divided, placed between the layers of Whatman filter paper no. 1, and compressed between glass plates under 2250 g. Calculation of weight loss after compression was expressed as percent drip loss. Cooking loss was measured by cooking the 25 g breast meat sample at 80 ${}^{\circ }$ C (75 ${}^{\circ }$ C internal temperature) for 45 min in a water bath (Honikel, 1998). The difference between breast meat sample weights before and after cooking was calculated as percent cooking loss.

### 2.9 Lipid and protein oxidation of liver, and fresh and freeze–thaw breast meat

Lipid peroxidation was measured in liver (at day 24 and 42) and breast meat (at day 42 in fresh and freeze–thaw breast fillet at 30, 60, and 90 d of storage) samples in terms of thiobarbituric acid reacting substances (TBARS). Liver samples were collected from all the slaughtered birds at day 24 ( n=20 /treatment; 80 in total) and day 42 ( n=20 /treatment; 80 in total) of the experiment. Approximately 200 g breast meat sample was collected from the left pectoralis major muscles of each chicken at day 42 ( n=20 /treatment; 80 in total). For the analysis of TBARS in fresh breast muscles, 10 g sample was cut and immediately processed. Remaining breast meat samples were divided into three equal portions and immediately frozen in liquid nitrogen and subsequently stored at - 20 ${}^{\circ }$ C in a deep freezer for measuring the TBARS in freeze–thaw breast meat after storage for 30, 60, and 90 d. Liver and breast meat samples were prepared for the quantification of TBARS using a modified Sørensen and Jørgensen method (Mielnik et al., 2006). Briefly, 10 g fresh or thawed breast meat was subjected to homogenization in 30 mL 7.5 % trichloroacetic acid solution at room temperature for 30 s at 15 000 rpm. The homogenate was filtered, 5 mL of the filtered homogenate was mixed with 5 mL thiobarbituric acid solution (0.02 mol L ${}^{-1}$ ) in a stoppered test tube, incubated for 35 min in a water bath at 100 ${}^{\circ }$ C, cooled in cold water for 10 min followed by the measurement (mg malondialdehyde kg ${}^{-1}$ ) of absorbance at 532 nm in a spectrophotometer (Shimadzu UV-1601, Kyoto, Japan) against the blank (5 mL thiobarbituric acid solution and 5 mL distilled water).

Protein oxidation was measured in breast meat only in terms of protein carbonyl and sulfhydryl groups in fresh (immediately after slaughter) and freeze–thaw breast meat after 90 d of storage. Carbonyl and sulfhydryl groups were measured in breast meat according to the method described by Srinivasan and Hultin (1997). The methods have been described briefly as follows.

In order to measure the carbonyl group, fresh or thawed breast meat samples were subjected to mincing followed by homogenization in a ratio of 1:10 ( w/v ) in pyrophosphate buffer (pH = 7.4) solution for 30 s. Homogenization was carried out in an ultra-turrax homogenizer. Pyrophosphate buffer was prepared using 2 mM pyrophosphate (Na ${}_{4}$ P ${}_{2}$ O ${}_{7}$ ), 10 mM tris-maleate, 100 mM potassium chloride (KCl), 2 mM magnesium chloride (MgCl ${}_{2}$ ), and 2 mM ethylene glycol tetraacetic acid solutions. Homogenates were separated into 0.1 mL, 1 mL 10 % triacetic acid was added, and precipitation of proteins in both aliquots was accomplished by centrifugation at 5000 rpm for 5 min. Supernatants were separated, and 1 mL 2 N hydrochloric acid (HCl) was added in one aliquot to measure the protein concentration, whereas an equal volume of 0.2 % ( w/v ) 2,4-dinitrophenylhydrazine in 2 N HCl was added in the other aliquot to measure the carbonyl group concentration. Afterwards, both the aliquots were allowed to incubate at room temperature for 1 h. Again, the aliquots were subjected to precipitation by adding 10 % triacetic acid, washed twice with 1 mL 1:1 ethyl alcohol/ethyl acetate solution, mixed, and centrifuged at 10 000 rpm for 5 min. The precipitates were dissolved by stirring in 1.5 mL sodium phosphate buffer solution (20 mM, pH = 6.5) containing 6 M guanidine hydrochloride. After dissolution, aliquots were centrifuged at 5000 rpm for 2 min followed by measurement of absorbance for protein concentration in a spectrophotometer (Shimadzu UV-1601, Kyoto, Japan) at 280 nm against bovine serum albumin standard. Concentration of carbonyl group was measured in the other aliquot by measuring the absorbance at a wavelength of 370 nm in the spectrophotometer.

Sulfhydryl group concentration was measured by the dissolution of minced fresh or thawed breast meat samples by shaking for 8 h at room temperature in 20 mL urea-SDS solution (pH = 7.4). Urea-SDS solution consisted of 8.0 M urea, 0.1 M phosphate, and 3 % SDS solution. Afterwards, 1 mL dissolved meat sample was transferred into an aliquot and 0.3 mL 5,5 ${}^{\prime }$ -dithiobis(2-nitrobenzoic acid) (DTNB) reagent prepared by the addition of 10 mM DTNB in 0.1 M phosphate buffer (pH = 7.4) was added into the aliquot and incubated for 15 min at room temperature. Finally, the absorbance of the sample was measured at 412 nm wavelength in a spectrophotometer (Shimadzu UV-1601, Kyoto, Japan) against the sample blank (1.0 mL phosphate buffer without DTNB) and reagent blank (distilled water only).

### 2.10 Antibody titer of infectious bursal disease virus

Serological evaluation of serum antibody titer (at day 42) against IBD was conducted with the help of commercial enzyme-linked immunosorbent assay (ELISA) kits (BioChek, Ascot, Berkshire, UK). Reading of the plates was accomplished using an ELISA plate reader (BioTek ELx800 Absorbance Microplate Reader, BioTek Instruments Inc., VT, USA).

### 2.11 Statistical analysis

Data were subjected to one-way analysis of variance (ANOVA) followed by Duncan's test as a post hoc test to assess the effect of treatments on all traits. Assumptions were made for significant differences at 95 % probability, whereas tendency was assumed when 0.05≤P≤0.1 . All the statistical evaluations were performed in the statistical software package SPSS (version 22.0, IBM Corp., NY, USA).

## 3 Results

### 3.1 Growth performance

Table 2 depicts the growth performance of broiler chickens in different groups. The study revealed a marked numerical increase in BW, BW gain, and FI of broilers fed diets supplemented with PFA alone or in combination with *B. licheniformis* compared to the control group; however, the differences were not statistically different. At the end of experiment, broiler chickens in PFA + *B. licheniformis* groups were more than 130 g per bird heavier than those in the control group. In addition, FI was numerically greater in PFA and PFA + *B. licheniformis* groups than the control group at the end of the experiment. Similarly, broiler chickens fed supplemental PFA and PFA + *B. licheniformis* had numerically better FCR than the control group.

**Table 2:** Growth performance of broiler chickens fed supplemental phytogenic feed additive alone or in combination with *Bacillus* *licheniformis*.

Days	Control	PFA ${}^{*}$	PFA + *Bacillus licheniformis*	SEM	P value
Body weight, g					
10	272	297	298	6.21	0.163
24	1217	1289	1262	17.52	0.248
42	2665	2798	2796	38.43	0.289
Body weight gain, g					
0–10	228	252	254	6.20	0.162
11–24	945	993	964	12.30	0.297
25–42	1448	1509	1533	24.64	0.376
0–42	2620	2753	2751	38.43	0.289
Feed intake, g					
0–10	232	232	236	6.39	0.962
11–24	1119	1176	1150	11.83	0.148
25–42	2714	2811	2814	50.39	0.690
0–42	4066	4219	4200	61.30	0.574
Feed conversion ratio					
0-10	1.02	0.93	0.93	0.03	0.242
11–24	1.19	1.19	1.19	0.01	0.903
25–42	1.87	1.86	1.84	0.02	0.622
0–42	1.55	1.53	1.53	0.01	0.543

${}^{*}$ PFA – phytogenic feed additives.

### 3.2 Carcass yield and characteristics

Absolute and relative weights of carcass, parts, and liver were not different among the groups, although an obvious numerical increase was noted in slaughter weight, absolute and relative weights of carcass, breast, and thigh muscles of broilers fed PFA alone or in conjunction with *B. licheniformis* (Table 3).

**Table 3:** Carcass yield and characteristics of broiler chickens fed supplemental phytogenic feed additive alone or in combination with *Bacillus licheniformis*.

Item	Control	PFA ${}^{*}$	PFA + *B. licheniformis*	SEM	P value
Slaughter weight, g	2776	2869	2873	25.72	0.226
Carcass weight, g	1959	2048	2050	20.65	0.123
Liver weight, g	52.75	50.80	50.20	1.09	0.616
Breast weight, g	728	784	781	10.73	0.052
Thigh weight, g	776	796	801	8.75	0.471
Carcass yield, %	70.54	71.34	71.32	0.20	0.166
Liver yield, %	1.89	1.77	1.75	0.03	0.117
Breast yield, %	37.08	38.26	38.04	0.25	0.111
Thigh yield, %	39.56	38.87	39.09	0.20	0.355

${}^{*}$ PFA – phytogenic feed additives.

### 3.3 Meat quality attributes

Dietary supplementation of PFA or PFA + *B. licheniformis* had no effect on breast meat quality of broiler chickens at slaughter and 24 h postmortem in comparison with the control group (Table 4).

**Table 4:** Meat quality of broiler chickens fed supplemental phytogenic feed additive alone or in combination with *Bacillus licheniformis*.

Item	Control	PFA ${}^{*}$	PFA + *B. licheniformis*	SEM	P value
At slaughter					
pH	6.38	6.41	6.45	0.03	0.686
$L^{*}$	48.72	49.14	50.37	0.43	0.275
$a^{*}$	1.90	2.35	2.00	0.14	0.414
$b^{*}$	6.42	7.55	7.24	0.21	0.078
24 h post-slaughter					
pH	5.87	5.91	5.91	0.01	0.438
$L^{*}$	54.05	54.88	54.99	0.34	0.458
$a^{*}$	4.04	3.52	3.09	0.19	0.128
$b^{*}$	10.28	10.17	9.77	0.23	0.632
Drip loss, %	8.47	7.86	8.72	0.31	0.526
Cooking loss, %	32.12	31.36	33.05	0.37	0.180

${}^{*}$ PFA – phytogenic feed additives.

### 3.4 Serum biochemistry

The serum biochemical profile of broiler chickens is presented in Table 5. Serum TP concentrations were similar across the groups at 24 and 42 d of the experiment. Broilers fed diets supplemented with PFA alone or in combination with *B. licheniformis* had greater serum albumin levels in comparison with the control group at day 24 ( P<0.001 ) and day 42 ( P<0.001 ) of the experiment. Serum AST levels remained unaffected across the treatments at day 24; however, supplemental PFA alone or in combination with *B. licheniformis* reduced ( P=0.004 ) the serum AST levels of broiler chickens compared to the control group at the end of experiment. Although serum GGT levels were lower in the PFA + *B. licheniformis* group than the control group at day 24 ( P=0.038 ), serum GGT levels were not different among the groups at day 42. A decrease in serum ALT levels of broilers was noted in PFA and PFA + *B. licheniformis* groups compared to those in the control group at day 24 ( P<0.001 ) and day 42 ( P<0.001 ).

**Table 5:** Blood biochemistry of broiler chickens fed supplemental phytogenic feed additive alone or in combination with *Bacillus licheniformis*.

Item	Control	PFA ${}^{*}$	PFA + *B. licheniformis*	SEM	P value
Day 24					
Total protein, g dL ${}^{-1}$	2.80	2.90	2.81	0.05	0.712
Albumin, g dL ${}^{-1}$	1.36 ${}^{b}$	1.53 ${}^{a}$	1.60 ${}^{a}$	0.03	<0.001
AST, IU L ${}^{-1}$	300.21	265.45	284.26	9.50	0.333
GGT, IU L ${}^{-1}$	15.95 ${}^{a}$	13.75 ${}^{\mathrm{ab}}$	13.25 ${}^{b}$	0.46	0.038
ALT, IU L ${}^{-1}$	21.65 ${}^{a}$	16.15 ${}^{b}$	17.00 ${}^{b}$	0.64	<0.001
Day 42					
Total protein, g dL ${}^{-1}$	2.96	3.23	3.08	0.06	0.220
Albumin, g dL ${}^{-1}$	1.31 ${}^{b}$	1.47 ${}^{a}$	1.59 ${}^{a}$	0.03	<0.001
AST, IU L ${}^{-1}$	346.50 ${}^{a}$	273.80 ${}^{b}$	279.15 ${}^{b}$	10.28	0.004
GGT, IU L ${}^{-1}$	18.75	16.65	18.25	0.62	0.356
ALT, IU L ${}^{-1}$	22.60 ${}^{a}$	18.20 ${}^{b}$	16.95 ${}^{b}$	0.57	<0.001

${}^{a,\;b}$ Means with different superscripts within the same row are significantly different. ${}^{*}$ PFA – phytogenic feed additives.

### 3.5 Jejunal histomorphometry

**Figure 1:**
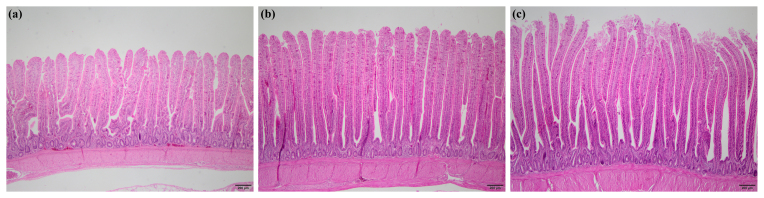
Representative photomicrographs of jejunal histomorphology of broiler chickens in **(a)** control, **(b)** phytogenic feed additive, and **(c)** phytogenic feed additive + *Bacillus licheniformis* groups.

Jejunal histomorphometry of broiler chickens at day 24 of the experiment is depicted in Table 6. Villus length, villus width, villus length : crypt depth ratio, and goblet cell number per villus of jejunum were greater in broilers in PFA and PFA + *B. licheniformis* groups than those in the control group ( P<0.001 , P=0.024 , P<0.001 , P=0.011 ). The control group had deeper crypts than PFA and PFA + *B. licheniformis* groups ( P=0.049 ). Surface area tended to increase ( P=0.098 ) in PFA and PFA + *B. licheniformis* groups in comparison with the control group. Representative photomicrographs of each group are shown in Fig. 1.

**Table 6:** Jejunal histomorphometry of broiler chickens fed supplemental phytogenic feed additive alone or in combination with *Bacillus licheniformis*.

Item	Control	PFA ${}^{*}$	PFA + *B. licheniformis*	SEM	P value
Villus length ( µ m)	1047.41 ${}^{b}$	1309.79 ${}^{a}$	1314.93 ${}^{a}$	25.30	<0.001
Crypt depth ( µ m)	186.97 ${}^{a}$	161.43 ${}^{b}$	154.08 ${}^{b}$	5.79	0.049
Villus width ( µ m)	145.58 ${}^{b}$	171.51 ${}^{a}$	182.12 ${}^{a}$	5.70	0.024
Villus length : crypt depth	5.89 ${}^{b}$	9.05 ${}^{a}$	8.98 ${}^{a}$	0.37	<0.001
Surface area (mm ${}^{2}$ )	0.58	0.60	0.70	0.02	0.098
Goblet cell number	130.75 ${}^{b}$	160.15 ${}^{a}$	176.45 ${}^{a}$	6.43	0.011

${}^{a,\;b}$ Means with different superscripts within the same row are significantly different. ${}^{*}$ PFA – phytogenic feed additives.

**Table 7:** Lipid peroxidation of liver, and lipid and protein oxidation of fresh and freeze–thaw breast meat of broiler chickens fed supplemental phytogenic feed additive alone or in combination with *Bacillus licheniformis*.

Item	Control	PFA ${}^{*}$	PFA + *B. licheniformis*	SEM	P value
Liver TBARS (mg malondialdehyde kg ${}^{-1}$ )					
day 24	1.94 ${}^{a}$	1.41 ${}^{b}$	1.50 ${}^{b}$	0.05	<0.001
day 42	1.84 ${}^{a}$	1.55 ${}^{b}$	1.57 ${}^{b}$	0.04	0.007
Breast Meat TBARS (mg malondialdehyde kg ${}^{-1}$ )					
Fresh	1.32	1.23	1.28	0.02	0.313
Freeze–thaw day 30	1.73 ${}^{a}$	1.42 ${}^{b}$	1.42 ${}^{b}$	0.04	0.002
Freeze–thaw day 60	1.87 ${}^{a}$	1.54 ${}^{b}$	1.56 ${}^{b}$	0.04	<0.001
Freeze–thaw day 90	1.88 ${}^{a}$	1.54 ${}^{b}$	1.56 ${}^{b}$	0.04	<0.001
Breast meat carbonyl (nmol mg ${}^{-1}$ protein)					
Fresh	53.06 ${}^{a}$	34.95 ${}^{b}$	35.23 ${}^{b}$	3.09	0.021
Freeze–thaw day 90	82.38 ${}^{a}$	50.82 ${}^{b}$	51.43 ${}^{b}$	4.49	0.003
Breast meat loss of sulfhydryl (nmol mg ${}^{-1}$ protein)					
Fresh	13.37	10.75	10.54	0.69	0.176
Freeze–thaw day 90	12.04	9.54	9.68	0.70	0.264

${}^{a,\;b}$ Means with different superscripts within the same row are significantly different. ${}^{*}$ PFA – phytogenic feed additives.

### 3.6 Lipid and protein oxidation of liver, and fresh and freeze–thaw breast meat

Dietary supplemental PFA and PFA + *B. licheniformis* lowered the lipid peroxidation by lowering the TBARS concentrations in liver at day 24 ( P<0.001 ) and day 42 ( P=0.007 ), and freeze–thaw breast meat after 30 ( P=0.002 ), 60 ( P<0.001 ), and 90 ( P<0.001 ) days of storage in comparison with the control group (Table 7). Dietary treatments had no effect on the TBARS in fresh breast meat and protein sulfhydryl groups in fresh and freeze–thaw breast meat. However, protein carbonyl groups in fresh and freeze–thaw breast meat samples were reduced ( P=0.021 and P=0.003 ) by the dietary supplementation of PFA alone or in combination with *B. licheniformis* compared to the control group.

### 3.7 Antibody titer against IBD

**Figure 2:**
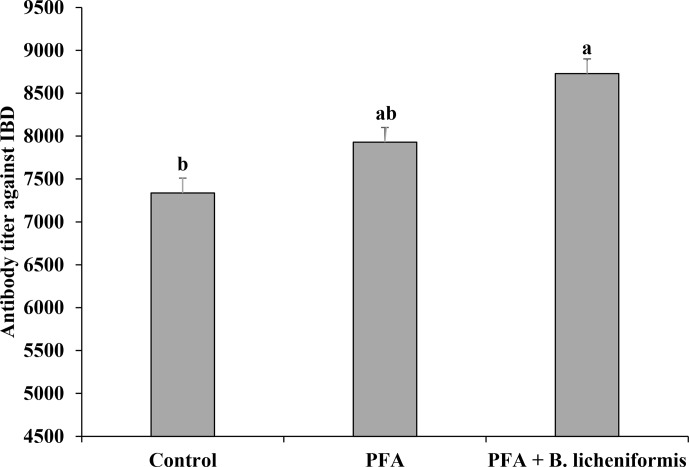
Antibody titer against infectious bursal disease (IBD) in broiler chickens fed supplemental phytogenic feed additive (PFA) alone or in combination with *B. licheniformis* (PFA + *B. licheniformis*). Broilers fed diets supplemented with PFA in combination with *B. licheniformis* had greater ( P=0.003 ) antibody titer against IBD than those fed basal diet only (control group). Different superscripts among the bars differ significantly.

Although dietary supplementation of PFA numerically increased the antibody titer against IBD compared to the control group, the difference was not significant (Fig. 2). Immune response against IBD was more pronounced ( P=0.003 ) in broiler chickens fed supplemental PFA along with *B. licheniformis* than those fed diets without any supplementation.

## 4 Discussion

The presence of various PFAs in the market and the increasing number of studies in this domain have complicated the evaluation and interpretation of the scientific studies. Over the past few years, this sector has seen a trend of mixing and blending of different phytogenics that has further added to this complication since different components in the blend may act differently, interact, and affect the efficacy of the components of individual phytogenic product. Therefore, PFA-related studies have reported inconsistent and inconclusive findings that require optimization in terms of their selection and usage regimens. Hence, the present study involved the use of PFA alone or in combination with *B. licheniformis*. Earlier studies have reported the effects of supplemental *B. licheniformis* alone in broiler chickens; therefore, the group with *B. licheniformis* was not included in this study.

### 4.1 Growth performance, and carcass yield and characteristics

BW, BW gain, FI, and FCR were numerically improved in broiler chickens in the PFA group compared to the control group. Many previous studies have reported no significant effect on the growth performance of broilers despite the differences in the composition of PFA used in those studies (Abildgaard et al., 2010; Kumar et al., 2010; Rizzo et al., 2010; Fascina et al., 2012; Abudabos and Alyemni, 2013; Hafeez et al., 2016; Ahsan et al., 2018; Ząbek et al., 2020). In contrast, other studies showed an improvement in the growth performance of broiler chickens in response to dietary PFA supplementation (Jamroz et al., 2005; Spernakova et al., 2007; Toghyani et al., 2010; Gheisar et al., 2015; Wati et al., 2015; Gheisar and Kim, 2018; Hassan et al., 2018; Movahhedkhah et al., 2019; Basit et al., 2020a, b). In our study, broiler chickens fed PFA in combination with *B. licheniformis* had greater BW, BW gain, FI, and better FCR than the control group despite the absence of any statistical difference. There are a limited number of studies describing the effect of dietary *B. licheniformis* on the growth performance of broiler chickens. Trela et al. (2020) reported an improvement in the growth performance of broiler chickens fed diets supplemented with *B. licheniformis*. Most of the studies have reported the effect of *B. licheniformis* in *Clostridium perfringens*-induced necrotic enteritis models (Knap et al., 2010; Zhou et al., 2016; Xu et al., 2018; Musa et al., 2019; Zhao et al., 2020). These studies reported the alleviation of deleterious effects of *C. perfringens* challenged broiler chickens, although the improvement in the growth performance in response to *B. licheniformis* was reported only by Zhou et al. (2016) and Zhao et al. (2020). In addition, there is a dearth of literature describing the combined use of PFA and probiotics. A study reported that simultaneous supplementation of PFA and a multi-strain probiotic enhanced the growth performance of broiler chickens (Mountzouris et al., 2015). The variation among the results of present and previous studies might be due to the differences in the composition of PFA and diets, probiotics, rearing conditions, and the presence of any stressor or challenge.

It was noted that broilers fed diets supplemented with PFA alone or in combination with *B. licheniformis* had numerically higher BW and BW gain, FI, and slightly better FCR than the control group. Similarly, this study showed that broiler chickens fed diets with PFA and PFA + *B. licheniformis* had greater slaughter, carcass, breast, and thigh weights and yields than those fed diets without supplementation, although the differences were not statistically different. These results are important from commercial perspective, although the statistical differences do not exist. This improvement in growth performance-related traits might be attributed to the beneficial effects of components of PFA and growth-promoting effects of *B. licheniformis*. The PFA used in our study was a homogeneous blend of capsaicin, glucosinolates, saponins, terpenes, and curcumin. The exact mechanism by which the improved growth performance occurred is not known. Capsaicin stimulates the digestive enzymes from pancreas and intestine (Platel and Srinivasan, 2004), and production of bile acids (Abdel Salam et al., 2005) in addition to the protection of gastric mucosa, thereby improving the digestibility of feed components. It also possesses antioxidant properties (Luqman and Razvi, 2006) and enhances the appetite (Yoshioka et al., 2001), which contribute to improve the growth of broiler chickens (Puvača et al., 2014, 2015). Glucosinolates at lower doses (subtoxic doses) act to protect against the oxidative insults to the cells (Belenli et al., 2016). Saponins exhibit a growth-promoting effect (Bafundo et al., 2021; Youssef et al., 2021) by increasing the intestinal mucosal permeability (Johnson et al., 1986), lowering the serum cholesterol, triglyceride, and glucose levels and improving the immune response (Bera et al., 2019), enhancing the litter quality of broiler chickens (Chaudhary et al., 2018), and by increasing the free-radical scavenging activity (Shi et al., 2014). Terpenes are also known to exert a growth-promoting effect (Cross et al., 2007) in addition to increasing antioxidant enzyme activity, secretions of digestive enzymes, gut morphology, and immune response of broiler chickens (Hashemipour et al., 2013; Ahsan et al., 2018). Curcumin is a polyphenolic compound that improves the growth of broiler chickens in addition to numerous biological functions (Rajput et al., 2013; Rahmani et al., 2018). Probiotics promote the growth of broiler chickens by stabilizing the gut health and ecosystem (Xu et al., 2018; Musa et al., 2019). It is speculated that the better growth performance, and carcass yield and characteristics in PFA and PFA + *B. licheniformis* groups might stem from one or more of these components through any or all of these pathways. However, in the present study, growth performance and carcass yield of broiler chickens in PFA and PFA + *B. licheniformis* was similar. It suggests that dietary supplementation of PFA promoted the growth of broiler chickens in both groups, leaving no further room for improvement by supplemental *B. licheniformis*. This enhancement of growth performance was reflected in the slaughter, carcass, breast, and thigh weights and yields.

### 4.2 Meat quality attributes

Supplemental PFA alone or in combination with *B. licheniformis* had no effect on meat quality of broiler chickens. Similar findings were reported by a previous study in response to dietary supplementation of PFA in poultry diets (Hong et al., 2012; Kirkpinar et al., 2014; Li et al., 2015; Yavaş and Malayoğlu 2019; Ao and Kim, 2020; Park and Kim, 2020). Generally, nutritional, and physiological states of muscle, and pre-slaughter conditions affect the meat quality due to their important role in the onset and development of rigor mortis (Zhao et al., 2012). Postmortem hypoxia prompts the anaerobic glycolytic pathway that utilizes the stored muscle glycogen to generate adenosine triphosphate (ATP) for utilization by muscles in addition to lactate that is accumulated in the muscle, thus lowering the pH of muscle (Duclos et al., 2007) to facilitate the conversion of muscle into meat. pH of meat is an important attribute of meat quality because it is indirectly associated with all other meat quality traits. Decline in meat pH in relation to the anaerobic glycolysis denatures the proteins that lowers the solubility of muscle proteins and positive and negative reactive groups responsible for binding the water (immobilized water) to the muscle proteins. As the protein denaturation takes place under influence of lowered pH, the opposite charges on the protein start neutralizing each other by attraction, which eventually reach an isoelectric point thereby losing their primary function to bind water. Subsequently, a shrinkage in myofibrillar space occurs due to the release of immobilized water that provides the opportunity to divalent sarcoplasmic cations (Ca ${}^{2+}$ and Mg ${}^{2+}$ ) to attenuate the anions present on adjoining protein chains. Eventually, electrostatic repulsion between the protein chains is diminished that releases the immobilized water, further reducing the water retention in the meat (Mir et al., 2017). Low pH results in greater drip loss and cooking loss or reduces the water-holding capacity, whereas higher pH favors the retention of water and thus increased water-holding capacity. It infers that meat pH determines the fate of meat quality in terms of taste, texture, tenderness, juiciness, color, drip loss, cooking loss, and water-holding capacity. In the present study, all the groups had the same pre-slaughter conditions in addition to meat pH that was within the optimum range (5.7–6.1) for poultry meat that do not display quality defects (Barbut, 1997). Therefore, meat quality attributes remained unaffected across the groups.

### 4.3 Serum biochemistry

Functionality of the liver is estimated by serum proteins and enzymes secreted from hepatocytes into the blood circulation. A decrease in serum TP and albumin and a surge in serum AST, ALT, and GGT levels occur in the case of stressful conditions, injury or damage to the liver, and disease conditions. As broiler chickens grow, an increase is noted in serum TP, albumin, ALT, AST, and GGT levels (Meluzzi et al., 1992) since growth is a stressful phenomenon in broiler chickens. Several proteins that carry out different functions like maintenance of blood volume, hormonal and drug transport, buffering (pH), and blood clotting are encompassed by serum TP in addition to albumin and immunoglobulins that are important in inflammatory and immune response (Melillo, 2013). In our study, dietary PFA alone or in combination with *B. licheniformis* enhanced the serum albumin and lowered the serum enzymes compared to the control group. These findings indicate that supplemental PFA alone or in combination with *B. licheniformis* had hepatoprotective effects in broiler chickens. Similar findings were reported in response to dietary capsaicin (Adegoke et al., 2018), curcumin (Adegoke et al., 2018; Rahmani et al., 2018), and terpenes (El-Ashram and Abdelhafez, 2020). However, previous studies described no effect of saponins (Chaudhary et al., 2018; Bera et al., 2019). In addition, no literature is available describing the effect of glucosinolates and *B. licheniformis* on the serum biochemistry of poultry. The exact mechanism by which dietary PFA and *B. licheniformis* increased the serum albumin levels and reduced the serum enzyme levels is not known. Rahmani et al. (2018) and Zhao et al. (2020) stated that improvement in serum biochemical profile of broiler is a manifestation of antioxidative effects of components of PFA and *B. licheniformis* that act by protecting the liver against hypoxia-induced free radicals that induce lipid peroxidation. We speculate that supplemental PFA alone or in combination with *B. licheniformis* might have shown their hepatoprotective effect by lowering the lipid peroxidation since liver TBARS were lower in PFA and PFA + *B. licheniformis* groups. Our findings related to serum biochemistry are supported by the lower lesion score that might have played its role to improve serum enzymes and proteins.

### 4.4 Jejunal histomorphometry

Optimal functioning of gastrointestinal tract of broiler chickens depends on the characteristic features that support larger surface area, which is dependent on longer and healthy villi, and shallower crypts. Shallow crypts indicate the slow or very low tissue turnover and a healthy intestine that might otherwise be deeper due to sloughing under normal conditions or owing to the inflammatory response. Deeper crypts contribute to the colonization of pathogens, as well as inefficient enzyme production, and consume more nutrients for tissue renewal due to faster and immature tissue turnover, thus leaving fewer nutrients for digestion, absorption, and growth of broiler chickens (Ahsan et al., 2016). In addition, bactericidal effect of mucin produced from an increased number of goblet cells per villus helps prevent the mucosal colonization of pathogens by binding with the pathogenic bacteria (Chacher et al., 2017). Previous studies have shown an improvement in the gut histomorphology of broiler chickens fed supplemental curcumin (Rajput et al., 2013; Rahmani et al., 2018), saponins (Bafundo et al., 2021; Youssef et al., 2021), terpenes (Ahsan et al., 2018), glucosinolates (Belenli et al., 2018), and other PFAs (Ząbek et al., 2020). In addition, probiotics have been known to improve the gut histomorphometry of broiler chickens (Zhou et al., 2016; Xu et al., 2018; Zhao et al., 2020). It is a well-known fact that PFAs and probiotics lower the pathogenic load by competitive exclusion and improve the intestinal immunity (Mountzouris et al., 2015). Therefore, the possible explanation for improved jejunal morphology in PFA and PFA + *B. licheniformis* groups might be the lower colonization of pathogenic microbes and immunomodulation in the intestine, thereby balancing the ecosystem that favored the lengthening and widening of villi, slower enterocytic turnover leading to shallow crypts, and increased goblet cells population, thus increasing the surface area of villi. Lower intestinal lesions evident from the lesion score boded well for the findings of jejunal histomorphometry of broiler chickens fed PFA or PFA + *B. licheniformis*.

### 4.5 Lipid and protein oxidation of liver, and fresh and freeze–thaw breast meat

Access to chicken meat is attributable to its availability at cheaper price and fast growth in a short lifespan in addition to public preference towards healthy eating impacting the consumption of animal proteins or meat. Consequently, nutritional and fatty acid composition of broiler diets has provided access to chicken meat with lower but healthy fat content consisting of omega-3 PUFAs. Proteins and PUFAs in chicken meat are prone to oxidation, especially the PUFAs that are highly susceptible to oxidation. Primary oxidation of PUFAs generates peroxides, whereas secondary oxidation produces TBARS and malondialdehyde (MDA) that are mutagenic, genotoxic, and carcinogenic besides induction of intracellular oxidative stress, membrane damage, and adduct formation (Reitznerová et al., 2017). Similarly, protein oxidation is a result of direct (by the reactive oxygen species) and/or indirect (by the products of oxidative insults) oxidation of sensitive amino acids subsequently modifying the protein function by fragmentation, aggregation, protein solubility, or decline in amino acid bioavailability. Consequently, an increase in the production of carbonyl groups along with increased loss of sulfhydryl groups is seen (Lund et al., 2011). Oxidation of fats and protein to oxidative functions of free radicals or reactive oxygen and nitrogen species results in poor quality of meat or meat products by altering the nutritive value, color, texture, and aroma. In the present study, lipid oxidation in liver and freeze–thaw breast meat after different days of storage was lower in groups fed PFA alone or in combination with *B. licheniformis*, while lipid oxidation was not different among the groups in fresh breast meat. In addition, PFA and PFA + *B. licheniformis* group had lower protein oxidation in fresh and freeze–thaw breast meat after 90 d of storage. Moreover, liver lesions were lower in PFA and PFA + *B. licheniformis* groups. The absence of any difference in the lipid oxidation of fresh breast meat might be attributed to the absence of any stressful condition as the broiler chickens were reared under standard management conditions without any application of physiological or environmental stress. Furthermore, the findings also suggest that supplemental PFA alone or in combination with *B. licheniformis* supported the oxidative stability of liver and breast meat during storage. The improvement in the oxidative stability of liver and breast meat might be attributed to the components of PFA or *B. licheniformis* as previous studies have reported the antioxidative activities of capsaicin (Oboh et al., 2007; Conforti et al., 2007), glucosinolates (Belenli et al., 2016, 2018), terpenes (El-Ashram and Abdelhafez, 2020), curcumin (Galli et al., 2020), and *B. licheniformis* (Zhou et al., 2016; Zhao et al., 2020).

### 4.6 Antibody titer against IBD

Immune response of broiler chickens vaccinated against IBD was better in the PFA + *B. licheniformis* group than the control group. Similar results were reported by Naseem et al. (2012) and Rehman et al. (2020) in response to multi-strain probiotics supplementation in broiler diets. Probiotics and PFA are known to modulate the humoral and cellular immunity in broiler chickens (see reviews Kabir, 2009; Yitbarek, 2015). It is speculated that dietary *B. licheniformis* might have improved the antibody titer against IBD by regulating the cytokines (Lammers et al., 2003). Other possible explanations might be the localized response in the intestine of broiler chickens in terms of activation of toll-like receptor (TLR) signalling pathway, regulation of mucosal immunity through cell-mediated immune response, promotion of intestinal barrier function, and enhancement of dendritic cell-induced hypo-responsiveness of T cells. The augmentation of TLR increases the capacity to recognize the components of pathogens, thus inducing the nuclear factor kappa of activated B-cell (NF- κ B)-dependent pathway that initiates the helper T cells to produce cytokines (Aalaei et al., 2019). It is believed that the increase in antibody titer against IBD was due to either *B. licheniformis* or synergism between PFA and *B. licheniformis* since PFA alone had no effect on the antibody titer against IBD.

## 5 Conclusions

The present study suggests that phytogenic feed additive alone or in combination with *B. licheniformis* might not improve the growth performance, carcass yield and characteristics, and meat up to a significant extent. However, the improvement in body weight, body weight gain, and feed intake, though not significant in this experiment, might be of interest for commercial settings. In addition, supplemental phytogenic feed additive and phytogenic feed additive + *B. licheniformis* may improve the serum biochemistry, jejunal morphology, and oxidative stability of liver and breast meat during storage by lowering the lipid and protein oxidation. Furthermore, combined supplementation of phytogenic feed and *B. licheniformis* may modulate the immune response of broiler chickens.

## Data Availability

The data are available from the corresponding author upon reasonable request.
